# Evaluating compliance with local and International Food Labelling Standards in urban Tanzania: a cross-sectional study of pre-packaged snacks in Dar Es Salaam

**DOI:** 10.1186/s12889-024-18488-9

**Published:** 2024-04-16

**Authors:** Hassan Rusobya, Fredirick Mashili, Ashabilan A Ebrahim, Zuhura Kimera

**Affiliations:** 1 School of Public Health, Muhimbili University of Health and Allied Sciences, United Nations Road, Upanga West, Dar es Salaam, Tanzania; 2 Department of Physiology, Muhimbili University of Health and Allied Sciences, United Nations Road, Dar es Salaam, Tanzania; 3 Department of Environmental and Occupational Health, Muhimbili University of Health and Allied Sciences, United Nations Road, Dar es Salaam, Tanzania

**Keywords:** Food labelling, Food labelling standards, Pre-packaged snacks, Front of package nutrition labelling

## Abstract

**Background:**

Urbanization influences food culture, particularly in low- and middle-income countries where there is an increasing consumption of processed and pre-packaged foods. This shift is contributing to a rise in non-communicable diseases. Food labelling standards are crucial for regulating manufacturing practices and helping consumers make healthy food choices. We aimed to assess the compliance of local and imported pre-packaged snacks with Tanzanian and international labelling standards in Dar es Salaam, Tanzania.

**Methodology:**

A cross-sectional study was conducted on 180 snack products. A checklist based on Tanzanian and Codex labelling standards was used to evaluate adherence. We also examined factors influencing adherence, such as product origin, price, category, purchase location, and package size.

**Results:**

The majority of the snacks demonstrated partial adherence to Tanzania (*n* = 97; 54%) and International (Codex) (*n* = 120; 67%) labelling standards. Imported products showed significantly better adherence to both Tanzanian (*n* = 46; 53%) and international (*n* = 42; 48%) standards. Notably, more than half (*n* = 110; 66.7%) of the products used English for labelling, and infrequently (*n* = 74; 41.4%) used the recommended World Health Organization Front-of-Pack Nutrition Labelling. Product category, origin, and package size were significantly associated with higher levels of international standard adherence (*p* < 0.05).

**Conclusion:**

The inadequate adherence to mandatory labelling standards and the scarce use of Swahili and FoPL highlight the need to strengthen labelling practices and potential challenges faced by consumers in understanding nutritional information. Thus, strengthening and emphasizing good labelling practices are urgently needed as we seek to address diet-related noncommunicable diseases.

## Introduction

Unhealthy dietary choices have become a global public health concern, leading to poor health outcomes and an increased risk of diet-related non-communicable diseases (NCDs) [1]. This has been influenced more by the widespread availability and marketing of less nutrient-dense foods [2, 3]. The increased accessibility of processed foods, specifically snacks, to supermarket shopping has been identified as a contributor to poor dietary habits [4], leading to an increase in overweight/obese individuals (BMI > 24.5 kg/m^2^) [5–7]. Labelling has been an important tool for informing consumers about the nutritional composition of foods, allowing them to make informed decisions about their food [8–10]..

In Tanzania, food labelling standards are regulated by the Tanzania Bureau of Standards (TBS) [11]. The agency is responsible for adopting and enforcing labelling standards established by the Codex Alimentarius Commission (CAC), an international organization for developing food standards [12]. However, individual nations have the autonomy to select what to include and what not to put on their standards, creating disparities in labelling standards among countries [13]. With the recent increase in international trade and with labels being considered a marketing tool, countries are recommended to adhere to all mandatory Codex requirements to ensure homogeneity in labelling and protecting consumers [7].

However, food product labels often lack completeness, and not all products conform to mandatory labelling requirements, with challenges being persistent in low-middle income countries (LMICs) [14–16]. Studies have highlighted deficiencies in the adherence of food products to international (Codex) food labelling standards [17, 18]. A recent study from Nigeria revealed that only 30% of food products comply with these standards [19]. This non-compliance with labelling standards hampers consumers’ ability to make informed choices, potentially contributing to unhealthy dietary practices [20]. The significance of proper food labelling is increasingly important because the consumption of pre-packaged foods has become increasingly common, particularly in sub-Saharan Africa, and has been associated with an increase in NCDs [15]. In response to this growing health concern, the World Health Organization (WHO) introduced the Global Action Plan for Prevention and Control of NCDs (2013–2020). Among the proposed strategies, the enforcement of food labels, including the Front of Pack Nutrition Labelling (FoPL), was suggested as a cost-effective approach to mitigate the impact of unhealthy diets [18]. Additionally, for Tanzania to participate in international trade, adhering to international labelling standards is not only necessary but also a key marketing strategy [21].

Despite the WHO recommendation and the given health and economic role of food labels, research evaluating adherence to food labelling among pre-packaged products in Tanzania is scarce. Therefore, this study aims to address this research gap by examining the extent of adherence to food labelling standards in Tanzania, encompassing both local and international standards. The investigation will focus on pre-packaged snacks available in Dar es Salaam, Tanzania’s largest city.

## Materials and methods

### Study design

This was a cross-sectional study involving the assessment of labels of 180 pre-packaged snacks collected from supermarkets and mini-stories in five districts in Dar es Salaam. Dar es Salaam, a center for trade with a wide range of manufacturing industries and an international harbour allowing for diverse availability and entry of pre-packaged foods, created a justifiable setting for conducting the study [22, 23].

### Inclusion and exclusion criteria

Pre-packaged snacks with and without labels falling under the Codex categorization of Bakery (e.g., Cakes, sweet biscuits, and pastries, other sweet bakery products), ready-to-eat savoury snacks Potato, cereal or starch-based (crisps, chips, and crackers), Confectionaries (chocolate, sweets, sweet toppings, energy bars, and desserts), and Sweetened Beverages (soda, energy drinks, milk, and dairy products) were eligible for the study [24]. Snacks with fainted fonts and those with prices above the 2,500Tsh average Tanzania daily individual earnings based on the National Bureau of Statistics (NBS) and Tanzania Annual Salary survey report [25, 26]were not included in the study.

### Sampling strategy

**Fig. 1:**
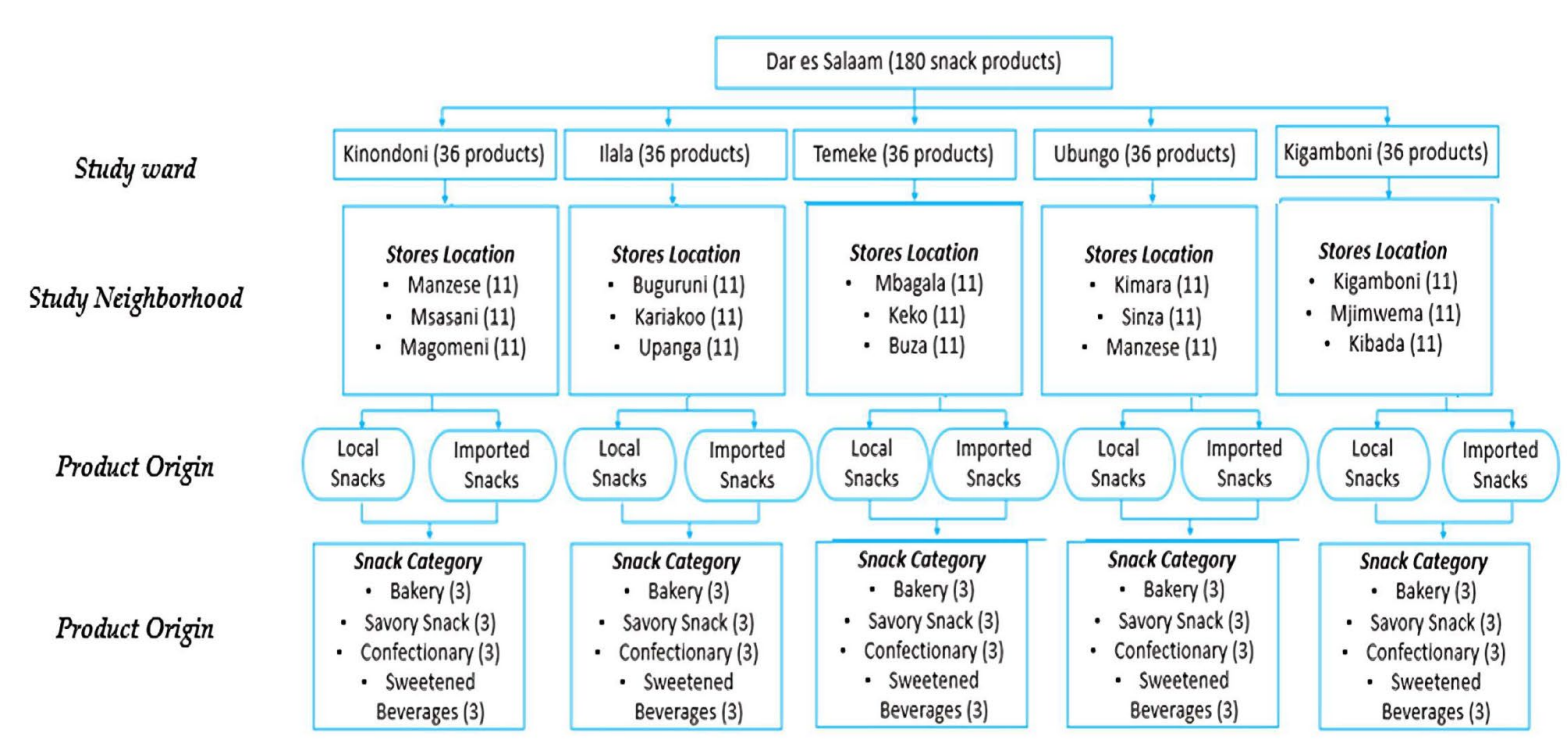
Framework illustrating how samples were collected including the study area and snacks category included

In this study, we utilized sampling protocols jointly developed by the WHO and Resolve to save lives for rapid assessment of trans-fatty acids (TFAs) in edible oils and foods [27, 28]. A complete list of registered supermarkets, mini-stores, and regularly purchased snacks within each ward was obtained from the ward marketing officer. Stratified cluster sampling was performed, where five districts of Dar es Salaam were considered strata included the Kinondoni, Ilala, Temeke, Ubungo, and Kigamboni districts. Each stratum was then clustered into wards, where three wards were randomly selected for the study. Within each ward, a total of ten mini-stores and supermarkets were randomly selected. Based on the regularity of purchase, simple random sampling was used for the selection of each snack category within shelves and rows of supermarkets and stores. Although this form of probability sampling is time-consuming, it is one of the most reliable methods for eliminating selection bias, as stated in a study by Maheshwari, 2017 [29]. The approach used is illustrated in Fig. 1.

### Data collection

An electronic checklist from Kobo Tool software [30] that included all the Tanzania and International mandatory labelling requirements was used. To allow for data cleaning and avoid missing information, the data were regularly transferred into Microsoft Excel software and cross-checked for consistency and completeness before they were exported to the Statistical Package for the Social Sciences (SPSS) for analysis.

Food products were categorized as either fully adherent or partially adherent to labelling standards. Although specific cut-off points are not available for categorizing product levels of adherence, categorization was designed to align with the number of Codex and Tanzania mandatory label requirements stipulated in a previous study [11, 24]. A product was considered fully adherent if it contained all ten (10) or thirteen (13) mandatory food labelling requirements from Tanzanian and international food standards, respectively, and partially adherent if it missed at least one of the mandatory food labelling requirements. Completeness of the nutritional information was inferred if a product was labelled with all seven essential nutrients (energy, carbohydrate, sugar, protein, fat, saturated fat, or salt/sodium) recommended by the Codex guidelines for nutritional labelling.

### Data analysis

The data were exported from Excel to the Statistical Package for the Social Science (SPSS) version 23 computer software for analysis. A p value of 5% indicated statistical significance. Categorical variables are presented as frequencies and proportions. Descriptive statistics were calculated to assess the proportion of local and imported snacks that adhered to both the Tanzania and international labelling standards and to determine which snack categories adhered more to the labelling standards. To assess the associations between product factors (product origin, snack category, purchase location, and package size) and the level of adherence to international (Codex) labelling standards, Pearson’s chi-square test was performed. Factors with a p value < 0.05 were further subjected to multivariable logistic regression analysis to assess their independent associations.

## Results

### Product characteristics

A total of 180 snacks from 165 stores and supermarkets were included in the study. The majority of the products were local snacks (*n* = 94; 52%), whereas an even number of snacks were assessed (*n* = 45; 27.3%) (Table 1).

**Table 1:** Description of product characteristics

Variables	Product Description	(*n* = 180)	(%)
Product Category	Bakery Snacks	45	27.3
	Savoury Snacks	45	27.3
	Confectionaries Snacks	45	27.3
	Sweetened Beverages Snacks	45	27.3
Product Origin	Local Product	94	52
	Imported Product	86	48
Purchase Location	Ubungo	36	20
	Temeke	36	20
	Ilala	36	20
	Kinondoni	36	20
	Kigamboni	36	20
Package Size	Large Package***	45	25
	Medium Package **	85	47.2
	Small Package *	50	27.8

***; (340 ≥ x ≤ 567 g); **; (170 ≥ x ≤ 283 g); *; (85 ≥ x ≤ 142 g)

### Adherence to labelling standards among snacks

**Fig. 2:**
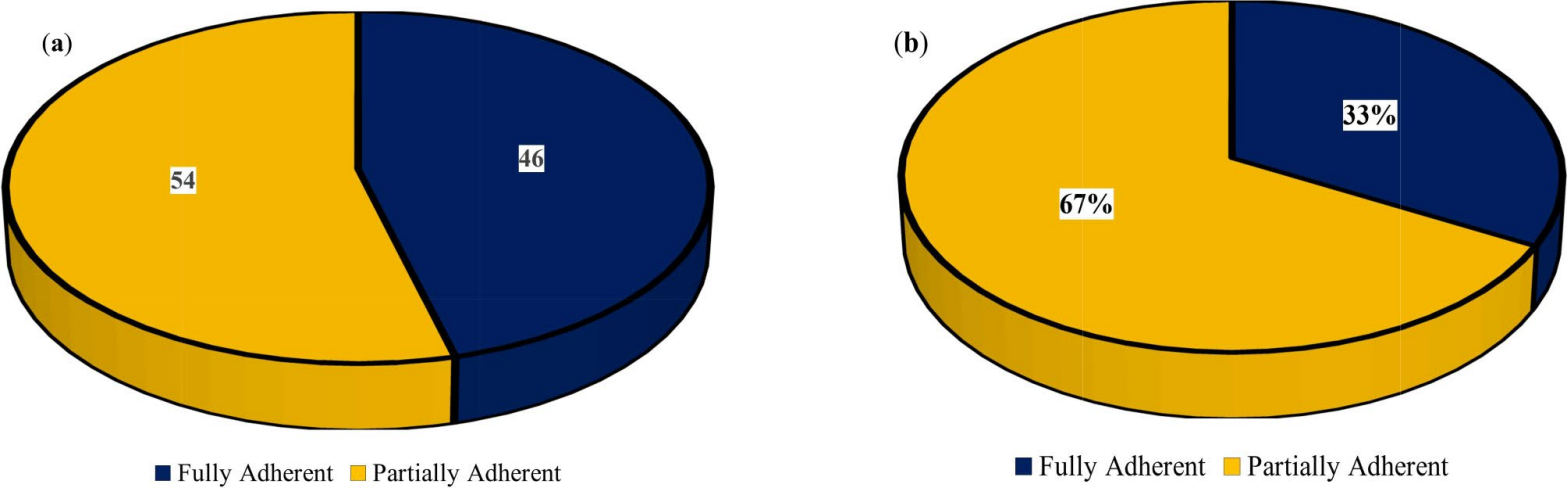
(**a**) Adherence to Tanzania’s mandatory labelling requirements; (**b**) Adherence to the International (Codex) mandatory labelling requirement

Almost half of the products (*n* = 82; 46%) were found to fully adhere to Tanzania’s labelling requirements (Fig. 2a), while only (*n* = 60; 33%) fully complied with international (Codex) requirements (Fig. 2b).

When examining the components of the labels, it was observed that the brand name (*n* = 180; 100%) was the most commonly available information on all the product labels. However, several essential components, such as the Quantitative Ingredient Declaration (QUID) (*n* = 85; 47%), instructions for use (*n* = 79; 44%) and the International Labelling Requirement of Allergen Information (*n* = 87; 48.5%), were notably missing.

### Adherence to labelling standards based on product origin

**Fig. 3:**
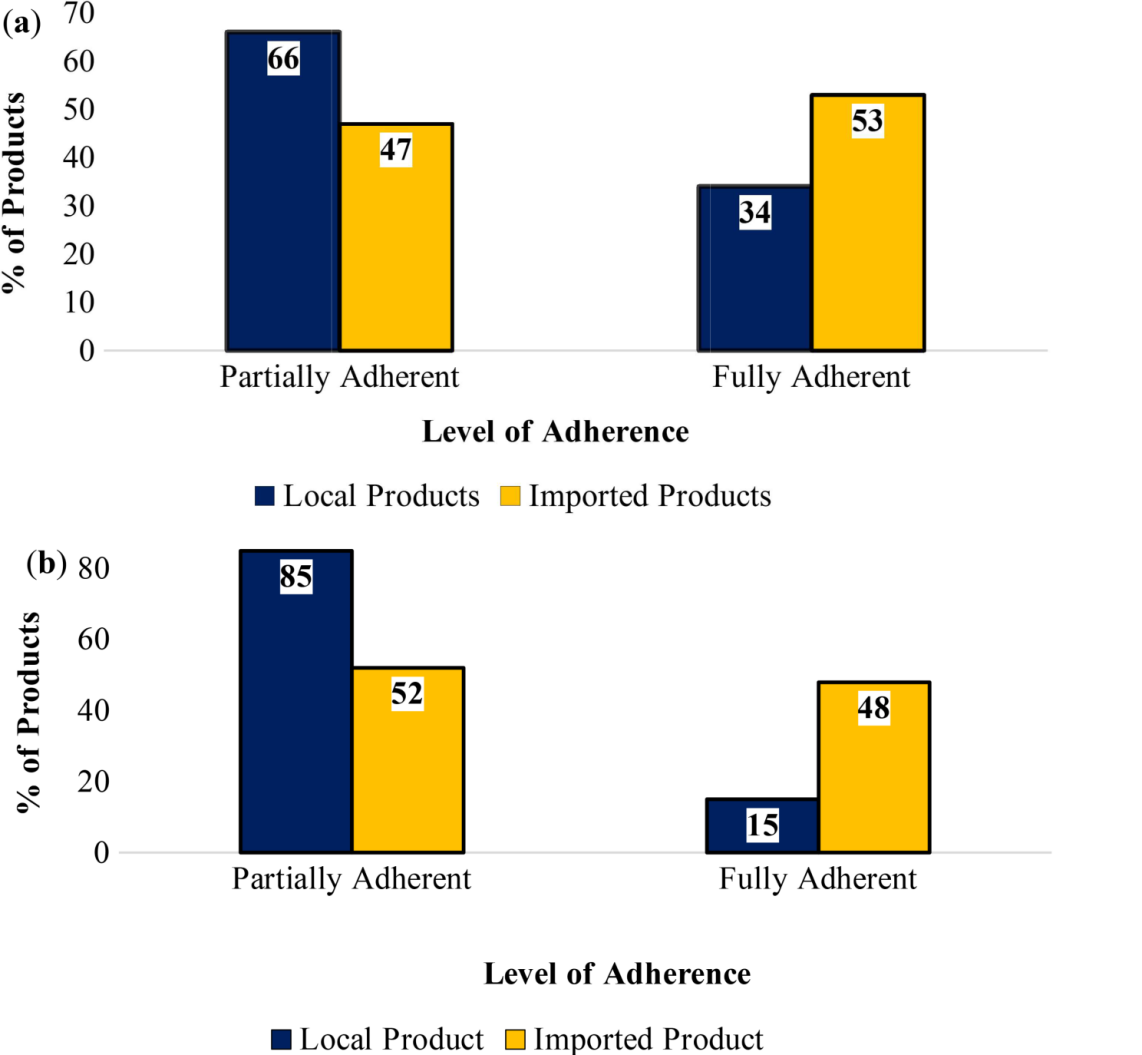
(**a**) Adherence to the Tanzania labelling standards among imported and locally produced snacks; (**b**) Adherence to the international labelling standards among imported and locally produced snacks

More than half of imported snacks (*n* = 46; 53%) were found to fully adhere to Tanzania’s labelling standards, whereas only (*n* = 32; 34%) of local products achieved full compliance (Fig. 3a). In terms of adherence to international labelling standards, (*n* = 42; 48%) of imported products fully met the international standards, greater than the mere (*n* = 14; 15%) of locally produced products that attained full adherence (Fig. 3b).

### Adherence to labelling standards based on product category

**Fig. 4:**
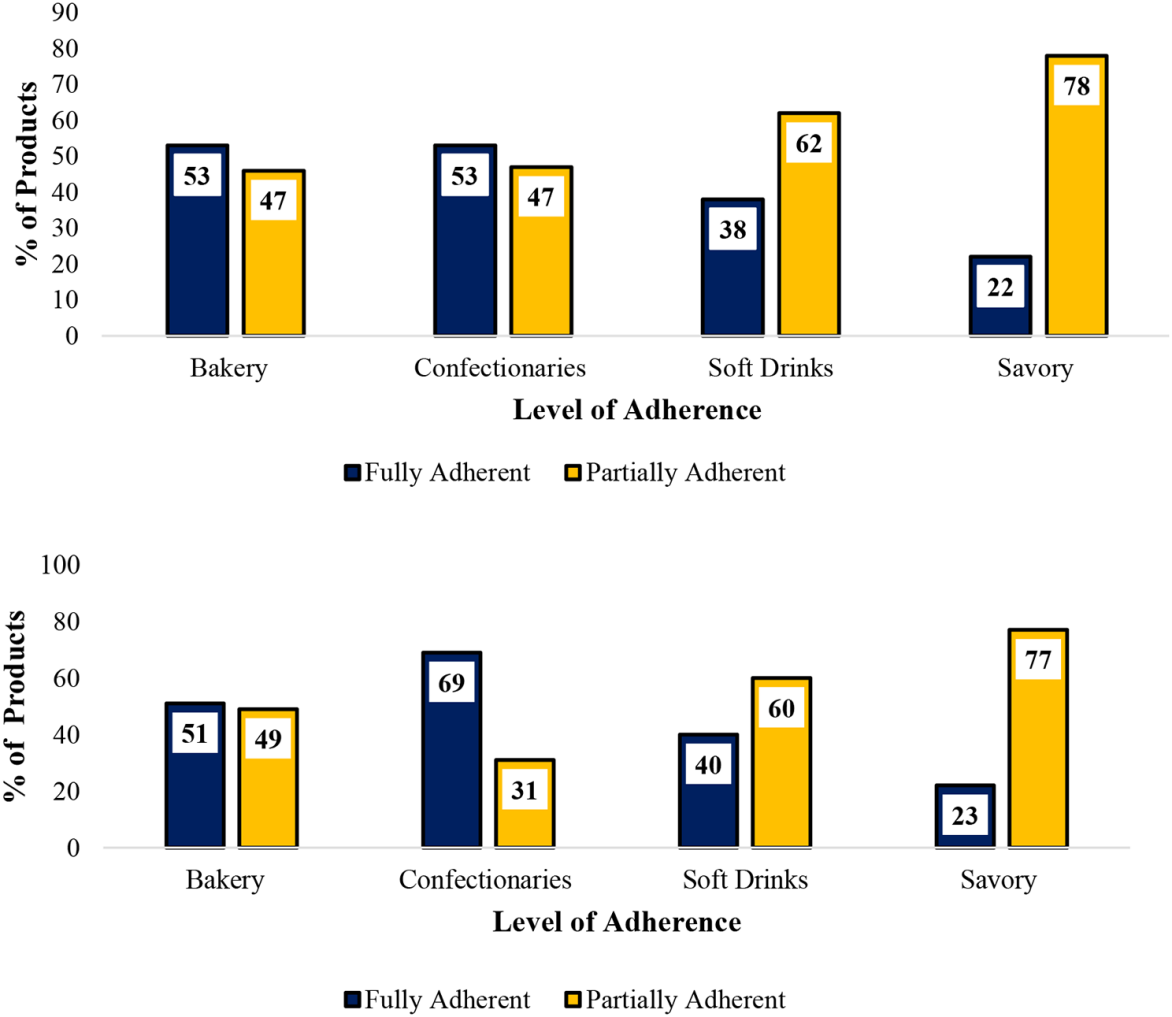
(**a**) Adherence to Tanzania Labelling Standards among Product Categories. (**b**) Adherence to International Labelling Standards among Product Categories

In terms of adherence to labelling standards based on product category, both baked snacks and confectioneries showed a high level of compliance with Tanzania’s labelling standard, with (*n* = 24; 53%) of products in each category registering full adherence (Fig. 4a). Confectioneries stood out in terms of adherence to the Codex standards, with (*n* = 31; 69%) of these products fully complying (Fig. 4b). In contrast, savory snacks were identified as the category with the lowest adherence to both the Tanzania and Codex labelling standards (Fig. 4a and b).

### Language and expiration date position

Out of the 180 products, more than half (*n* = 110; 61.1%) used the English language for labelling. Similarly, among the 94 local products, only (*n* = 14; 14.8%) used the Swahili language. However, the use of English in combination with other languages (Arabic, Greek, Turkey, etc.) was observed (*n* = 39; 41.5%), most being imported products. With respect to the inclusion of expiration (best before) dates, all the products in the study had this information. However, the positioning of the expiration date on the packaging varied among the products. Approximately (*n* = 103; 57%) of the products highlighted the expiration date prominently at the top or in front of their packaging.

### Adherence to the nutrition declaration

**Fig. 5:**
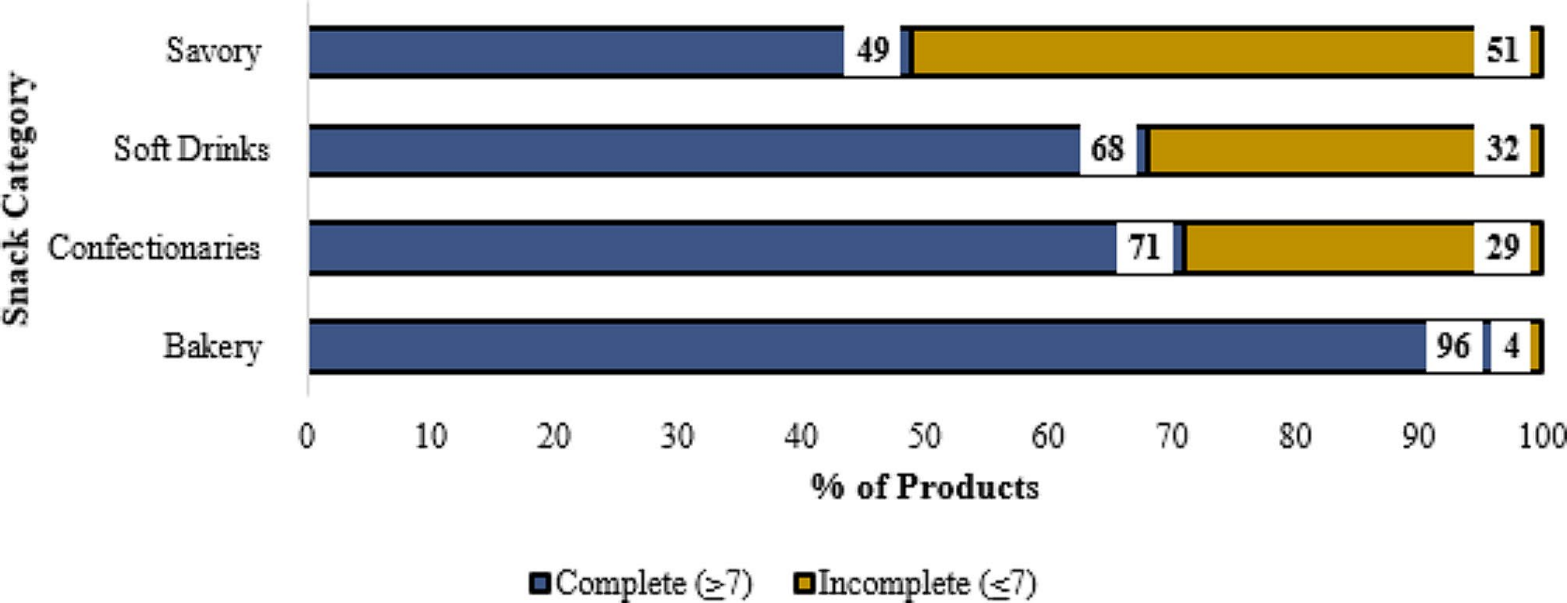
Nutrition declaration status among snack categories.

Of the total products studied, the majority, (*n* = 113; 62.7%), had complete nutritional data. More than more than three-quarters of imported products (*n* = 83; 86%) completed their nutritional declarations, with less than half (*n* = 30; 42.3%) of the local products fully presenting that information (Fig. 5).

When comparing different snack categories, (*n* = 43; 96%) of the bakery snacks had complete nutritional information. On the other hand, the lowest proportion of savoury snacks (*n* = 22; 49%) presented complete nutritional information.

### Nutrition profiling schemes

**Fig. 6:**
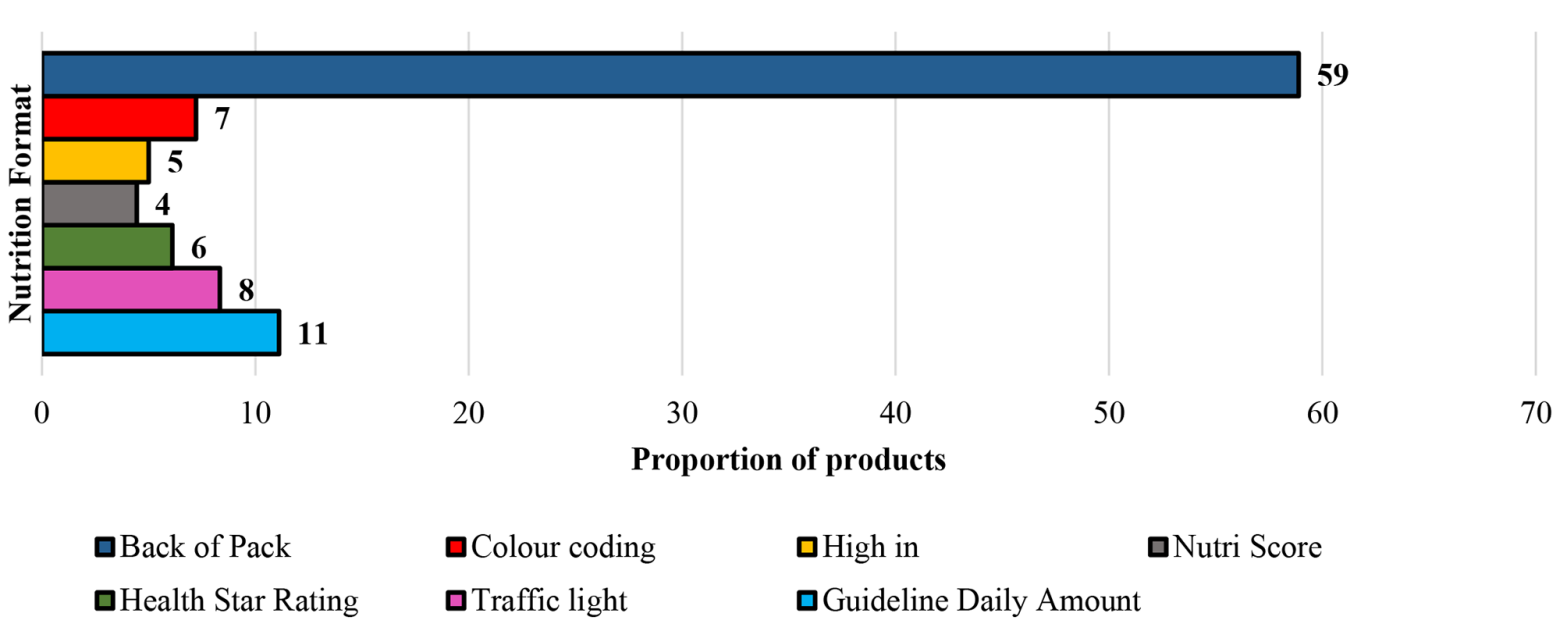
Pre-packaged products with various front-of-pack labelling schemes

A greater number of product labels (*n* = 106; 59%) presented nutritional information using the traditional Back of Pack nutrition Labelling (tabular) format. In contrast, a smaller proportion (*n* = 74; 41%) employed one or more of the WHO recommended Front of Pack nutrition Labelling (FoPL) formats such as colour coding, High in, and Nutri score. Notably, the majority of products identified using FoPL labelling (*n* = 115; 64%) were imported products (Fig. 6).

### Product-related factors associated with adherence to international labelling standards

Product category (p value < 0.024), product origin (p value < 0.001), and package size (p value < 0.016) exhibited statistically significant associations with adherence to the Tanzania labelling standards. After adjusting for confounders, package size (p value < 0.005) and product origin (p value < 0.002) demonstrated an independent association with adherence to international labelling standards. Specifically, the imported ([aOR] = 3.24; 95% [CI] = 2.84–7.52) and medium-sized packages ([aOR] = 4.09; 95% [CI] = 1.23–10.9) were more fully adherent to the International Standard than were the local and small-sized packages, respectively (Table 2).

**Table 2:** Product-related factors associated with adherence to international labelling

Variables	International(Codex) Adherence Status		Bivariate analysis	Multivariate analysis	
	Fully Adherent (*n* = 180)	Partially Adherent (*n* = 180)	P value	aOR (95% CI)	P value
**Product Category**					
Savoury	24	21		3.29 (1.20–9.01)	0.05
Sweetened beverages	17	28	0.02*	1.04 (0.40–2.72)	0.92
Confectionaries	24	21		0.75 (0.32–1.76)	0.52
Bakery	10	35		**Reference**	
**Product Origin**					
Imported Product	50	44	0.001*	0.31 (0.16 -0.64)	0.002*
Local Product	25	46		**Reference**	
**Package Size**					
Large	22	16		1.76 (0.57–5.38)	0.319
Medium	36	63	0.016*	4.09 (1.23–10.9)	0.005*
Small	17	11		**Reference**	

*Product factors that showed an association with the level of adherence (p value < 0.05); aOR, adjusted odds ratio

## Discussion

Food labels serve as critical tools for promoting healthier food choices, contributing to global efforts to promote healthier food choices, enhance food safety, and mitigate the escalating crisis of NCDs. Our study, while focused on Tanzania, taps into a universal challenge, reflecting a scenario prevalent in many parts of the world, particularly in low- and middle-income countries that are undergoing rapid urbanization and dietary transitions. Our findings offer valuable insights into the complexities and challenges of implementing effective food labelling practices—a concern that resonates globally—given the WHO drive for mandatory labelling and the front of nutrition package labelling (FoPL) as key strategies in the worldwide fight against NCDs [18].

Our findings indicate notable adherence to Tanzanian labelling standards among snack products, although adherence to international (Codex) standards was less prevalent, echoing similar observations in other African regions. Kokobe et al. [31] in Ethiopia and Nigeria [19] also reported higher compliance among imported products than among local products. This discrepancy highlights a critical need for increased investment in labelling education and awareness, particularly targeting local micro food producers.

The variation in adherence across snack categories, with poorer adherence for savoury snacks, as noted in our study, is similar to findings from a meta-analysis across European countries [32]. This disparity is likely due to differences in manufacturing practices, with larger industries typically adhering more strictly to regulations [33]. This finding suggests the need for more robust regulation and support for smaller-scale producers, who often contribute to the savoury snacks category.

Another significant concern observed in our study was incomplete adherence to mandatory labelling requirements, particularly the lack of essential information such as ingredient lists, usage instructions, and allergen information. While the missing information might not be viewed as particularly important, this gap in labelling poses a health risk, as illustrated by Dumoitier et al. [34] and a Canadian study linking missing allergen information to increased accidental allergic reactions [35].

We also found that the majority of the labels used English, with limited use of Kiswahili, raising concerns about consumer comprehension. Given that a substantial portion of Tanzania’s population is more proficient in Kiswahili, these findings underscore the importance of language translation and localization in labelling, as emphasized in most studies [36, 37]. Additionally, our analysis revealed that FoPL, despite being recommended by the WHO, was not widely employed. This finding contrasts with studies in regions such as the United Kingdom, where FoPL is more commonly used [38, 39]. The inadequate use of FoPL, which is not a mandatory requirement of the Codex Commission, allows manufacturers to conceal nutritional information, especially to consumers who are not literate enough to understand the technicality of what is present.

Factors such as product origin and package size significantly influenced adherence to labelling standards, compliance with imported products, and compliance with large packages compared to local and small packaged snacks, respectively. This finding aligns with research conducted in Malaysia [40, 41], suggesting that individuals with smaller packages and locally produced snacks tend to have lower compliance rates. This emphasizes the need for stricter scrutiny of labelling practices, particularly for local and smaller packaged products.

However, our study is not without limitations. Despite sampling the most popular snacks, the focus on only four snack categories may not fully represent the diversity of snacks available, highlighting the need for broader research. Additionally, the cross-sectional nature of the study limits our ability to capture dynamic trends or changes in labelling regulations and practices, which are currently common.

## Conclusions

In conclusion, while Tanzania shows promising progress in food labelling practices, considerable efforts are still needed to achieve full compliance with mandatory standards, particularly among locally produced products. Emphasis on using Kiswahili for labelling and enforcing policies for FoPL implementation is crucial. Strengthening regulatory frameworks and enhancing support for small-scale producers will be key to improving food labelling standards, helping consumers make informed healthier food choices and ultimately contributing to the reduction of noncommunicable diseases.

## Data Availability

The data presented in this study are available upon request from the corresponding author.

## Abbreviations

BMI: Body Mass Index
CAC: Codex Alimentarius Commission
FoPL: Front of Pack Labelling
LMICs: Low Middle-Income Countries
NCDs: Non-Communicable Diseases
WHO: World Health Organization
TBS: Tanzania Bureau of Standards
