# Publisher Correction: Evaluating compliance with local and International Food Labelling Standards in urban Tanzania: a cross-sectional study of pre-packaged snacks in Dar Es Salaam

**DOI:** 10.1186/s12889-024-19054-z

**Published:** 2024-06-07

**Authors:** Hassan Rusobya, Fredirick Mashili, Ashabilan A Ebrahim, Zuhura Kimera

**Affiliations:** 1 School of Public Health, Muhimbili University of Health and Allied Sciences, United Nations Road, Upanga West, Dar es Salaam, Tanzania; 2 Department of Physiology, Muhimbili University of Health and Allied Sciences, United Nations Road, Dar es Salaam, Tanzania; 3 Department of Environmental and Occupational Health, Muhimbili University of Health and Allied Sciences, United Nations Road, Dar es Salaam, Tanzania

**BMC Public Health (2024) 24:1062**

10.1186/s12889-024-18488-9

In the original publication of this article figure 6 was not fully visible due to an error in the publication process. The original publication has been updated to rectify this error. The publisher apologizes to the authors & readers for the inconvenience caused by this.

